# Entropy Affects the Competition of Ordered Phases

**DOI:** 10.3390/e20020115

**Published:** 2018-02-10

**Authors:** Balázs Király, György Szabó

**Affiliations:** 1 Department of Theoretical Physics, Faculty of Natural Sciences, Budapest University of Technology and Economics, Budafoki út 8, H-1111 Budapest, Hungary; 2 Institute of Technical Physics and Materials Science, Centre for Energy Research, Hungarian Academy of Sciences, P.O. Box 49, H-1525 Budapest, Hungary

**Keywords:** evolutionary games, lattice systems, potential games, phase transitions

## Abstract

The effect of entropy at low noises is investigated in five-strategy logit-rule-driven spatial evolutionary potential games exhibiting two-fold or three-fold degenerate ground states. The non-zero elements of the payoff matrix define two subsystems which are equivalent to an Ising or a three-state Potts model depending on whether the players are constrained to use only the first two or the last three strategies. Due to the equivalence of these models to spin systems, we can use the concepts and methods of statistical physics when studying the phase transitions. We argue that the greater entropy content of the Ising phase plays an important role in its stabilization when the magnitude of the Potts component is equal to or slightly greater than the strength of the Ising component. If the noise is increased in these systems, then the presence of the higher entropy state can cause a kind of social dilemma in which the players’ average income is reduced in the stable Ising phase following a first-order phase transition.

## 1. Introduction

Multi-player evolutionary games are simple models that can be employed to study a wide range of systems across several diverse disciplines [1,2,3,4,5,6,7,8,9]. In these mathematical models identical players are located at the nodes of a network structure (e.g., at the sites of a lattice), they repeatedly play the same two-player game against their neighbors, and are allowed to change their strategy according to some evolutionary update rule. The pair interaction is defined by an n×n payoff matrix [10] if the players can choose among *n* options (henceforth called strategies). The large number of payoff parameters causes difficulties in systematic investigations and in the classification of possible behaviors as well. These difficulties can be reduced by recognizing that two games are equivalent if their payoff matrices can be transformed into each other by exchanging some strategy labels.

The linear decomposition of payoff matrices [11,12,13,14] can easily reveal such symmetry relations, provides a classification scheme for payoff matrices, and allows us to distinguish fundamentally different types of elementary games [15,16]. This approach generalizes the two-strategy coordination game that enforces players choosing the same strategy. These latter models are equivalent to the kinetic Ising model if n=2 and the logit rule controls the evolution [17,18,19,20,21,22,23,24,25,26,27]. For *n* strategies, generalized coordination games are composed of two-strategy coordination subgames between all possible strategy pairs [16]. When the logit rule is used [28,29,30], some lattice versions of these models become equivalent to spin systems or lattice gas models (e.g., Potts models [31] or the Ashkin-Teller model [32]) that exhibit thermodynamic behavior [33,34,35]. In “real” coordination games the maximal value of the payoff matrix is located in its main diagonal, and the corresponding strategy is preferred by all players. Consequently, the strategy distribution observed at low noises can be considered as an ordered phase that transforms into a disordered strategy arrangement if the noise is increased.

So far, we have tacitly considered cases where the maximal payoff is unique or degenerate in such a way that equivalent phases possess similar features and symmetries at low noises. What happens when the latter conditions are not satisfied? Which of the possible equilibria will be preferred? For equal average payoffs in the ordered strategy arrangements, it will be shown that these systems select one of those ordered phases that have higher entropy. Furthermore, entropy can have a similar role even when the payoffs of the ordered phases are tuned away from being equal.

In this paper we investigate the stabilization effects of entropy in a five-strategy coordination game where the subgames of the first two and the last three strategies are identical to a ferromagnetic Ising and a three-state Potts model, respectively. It is also assumed that the players receive zero income if they choose strategies belonging to different subgames. Using Monte Carlo simulations and cluster variation methods, we show that one of the “Ising phases” is stabilized by its higher entropy at low noises if the payoffs are identical in all five ordered strategy arrangements. Furthermore, this system undergoes an Ising-type phase transition when the noise is increased. Evidently, any small extra payoff in the “Potts phase” can stabilize it in the low noise limit. In the rest of the paper we describe the model in detail and quantitatively study its phase transitions for several payoff values.

## 2. The Model and Its General Features

Throughout this paper we study evolutionary games on an N=L×L-site square lattice with periodic boundary conditions [6,13,36]. There is one player located at each site, all of whom repeatedly play the same two-player game with their four nearest neighbors and may choose one of *n* available pure strategies to play in all four games. If we represent these strategies with the *n* Cartesian unit vectors of an *n*-dimensional Euclidean space, then the income of the player at site *x* can be calculated as

(1) $${\tilde{u}}_{x}\left(s_{x}\right)=\sum_{\delta }s_{x}\cdot As_{x+\delta }.$$

Here $s_{x}$ denotes the strategy chosen by the player at site *x*, the summation runs over this player’s nearest neighbor sites (x+δ), and the element $A_{ij}$ of the n×n payoff matrix is the income of the player playing the *i*-th strategy against his/her opponent selecting the *j*-th strategy [10].

This paper is focused on the study of a five-strategy game in which subgames associated with its first two (henceforth strategy 1 or 2) and last three strategies (labelled by numbers 3, 4, and 5) are an Ising and a three-state Potts model, respectively. Opposing players whose strategies belong to different subgames both receive zero payoff. We note again that we use pure coordination-type games for which the sum of payoffs is zero in each row and column of the payoff matrix. Consequently, the elements of our Potts payoff matrix are shifted by a uniform irrelevant constant in comparison with the standard diagonal Potts payoff matrix [31,37]. If the Ising subgame’s payoff rewarding coordination is chosen to be the unit of currency and α≥0 is the relative strength of the Potts subgame, then the payoff matrix takes the following form:

(2) A=−1−1−0−0−0−1−1−0−0−0−0−0−α−α2−α2−0−0−α2−α−α2−0−0−α2−α2−α.

This payoff matrix contains a coordination-type (Ising) interaction between strategies 1 and 2 with unit strength while the strategy pairs (3,4), (3,5), and (4,5) are coordinated similarly with a strength of α/2 [16]. Our analysis is restricted to α>0.

The present game is a potential game because its payoff matrix is symmetric ($A_{ij}=A_{ji}$), and its corresponding potential matrix is V=A [13]. In this evolutionary game the potential U(s) of the whole system, a function of strategy configuration $s=\{s_{x}\}$, can be expressed as a sum of the pair potentials. Namely,

(3) $$U\left(s\right)=\frac{1}{2}\sum_{x,\delta }s_{x}\cdot Vs_{x+\delta }.$$

This quantity resembles the negative potential energy (Hamiltonian) of a physical system. Maximal total payoff is achieved if all players choose either strategy 1 or 2 (called an Ising phase) if 0<α<1. For α>1, however, the total payoff reaches its maximum in one of three ordered Potts phases when all players uniformly choose strategy 3 (or 4 or 5).

If the evolution of strategy profile s is controlled by the so-called logit rule, then a randomly selected player may change his/her strategy $s_{x}$ to any available pure strategy $s_{x}^{\prime }$ with payoff-dependent probability

(4) $$w\left(s_{x}^{\prime }\right)=\frac{e^{{\tilde{u}}_{x}\left(s_{x}^{\prime }\right)/K}}{\sum_{s_{x}}e^{{\tilde{u}}_{x}\left(s_{x}\right)/K}},$$

where the parameter *K* quantifies external noise (or temperature) affecting the decision-making abilities of players. This rule exponentially favors higher payoffs, provided that the opponents do not modify their strategies [28,29,30,35].

The main advantage of the application of the logit rule is that the repetition of the above elementary strategy changes will drive potential games into the Boltzmann distribution [33,34] when strategy configuration $s=\{s_{x}\}$ is realized with probability

(5) $$p\left(s\right)=\frac{e^{U(s)/K}}{\sum_{s^{\prime }}e^{U(s^{\prime })/K}}.$$

The macroscopic behavior of this system can be quantified by the average proportions $\rho_{i}$ of players following the *i*th strategy (i=1,2,3,4,5). The general features of this evolutionary game share many similarities with those of the constituent Ising and Potts models. At high temperatures (*K*) the system evolves into a disordered state in which strategies are chosen by almost equal shares of the population, or more precisely, all five strategy frequencies approach 0.2 when *K* goes to infinity. On the other hand, strategy choices are homogenized in the low-noise limit, and we find the system in one of the competing ordered phases. When α>1, the Potts phase prevails in which all players follow one of the Potts strategies (e.g., strategy 3, 4, or 5). In the following, we will assign label 3 to the strategy that forms the Potts phase as K→0. In the other case (0≤α≤1) one of the Ising strategies dominates the low-temperature regime, which we will call strategy 1 without any loss of generality.

## 3. Methods

### 3.1. Mean-Field and Pair Approximations

Mean-field and pair approximations belong to the family of cluster variation methods [13,38,39,40]. In translation invariant lattice systems the mean-field approximation describes states with one-site probabilities $p_{1}\left(i\right)=\rho_{i}$ of finding strategy *i* at any site. Obviously, these probabilities are normalized, that is, $\sum_{i}p_{1}\left(i\right)=1$. Similarly, pair approximation employs two-site probabilities $p_{2}\left(i,j\right)$ of strategy *i* and strategy *j* occupying neighboring sites. Both methods evaluate independent equilibrium configuration probabilities as extremal points of a thermodynamic potential Φ that is analogous to Helmholtz free energy:

(6) Φ=U+KS

where U is the average value of the potential [$U=\sum_{s}p\left(s\right)U\left(s\right)$], and S is the (Gibbs) entropy [$S=-\sum_{s}p\left(s\right)\ln p(s)$] of the system.

On a square lattice, Φ can be estimated by

(7) $$\Phi^{\left(1\right)}=2N\sum_{i,j}p_{1}\left(i\right)p_{1}\left(j\right)A_{ij}-NK\sum_{i}p_{1}\left(i\right)\ln p_{1}\left(i\right)$$

at the level of mean-field approximation, while its two-site-approximated value is given by

(8) $$\begin{array}{c} \Phi^{\left(2\right)}=2N\sum_{i,j}p_{2}\left(i,j\right)A_{ij}-2NK\sum_{i,j}p_{2}\left(i,j\right)\ln p_{2}\left(i,j\right)+\;3NK\sum_{i}p_{1}\left(i\right)\ln p_{1}\left(i\right), \end{array}$$

where the one-site occupation probabilities can be evaluated via so-called compatibility conditions:

(9) $$p_{1}\left(i\right)=\sum_{j}p_{2}\left(i,j\right)=\sum_{j}p_{2}\left(j,i\right).$$

We used different methods to find the extremal points of the approximated thermodynamic potentials. In the mean-field case a direct numerical approach was employed: At extremal points the partial derivatives of the free energy with respect to one-site probabilities should vanish, that is,

(10) $$\frac{\partial \Phi^{\left(1\right)}}{\partial p_{1}\left(i\right)}=0.$$

We have to keep in mind the nonnegativity and normalization properties of probabilities as well. These equations can be solved numerically. Our results are presented in Section 4. This technique can be straightforwardly extended to pair approximation. Due to the increased number of independent variables and equations, however, finding competing solutions becomes prohibitively harder. That is why we turned to analyzing the dynamical pair approximation equations of motion [13,41] that dictate the time evolution of two-site probabilities $p_{2}\left(i,j\right)$ under the logit rule. We have found that using this approach with initial states inspired by mean-field and Monte Carlo results gives easier access to metastable solutions of the pair approximated model.

### 3.2. Monte Carlo Simulations

We also employed Monte Carlo simulations [42] to numerically investigate the stationary states. The simulations were carried out on a square lattice of N=L×L sites, with periodic boundary conditions. The average frequencies of the strategies ($\rho_{i}$) were evaluated by averaging over a sampling time $t_{s}$ after waiting for a relaxation time $t_{r}$. The linear size of the system was varied from L=400 to 3,000, relaxation and sampling times ranged from $10^{4}$ to $10^{6}$ Monte Carlo steps. Each player received one chance (on average) to change his/her strategy during each Monte Carlo step. The larger system sizes and longer run times were used near phase transitions in order to dampen the effects of diverging fluctuations, correlation lengths, and relaxation times.

The competing Ising and Potts phases were studied using ordered initial states (homogeneously prepared Ising or Potts states) due to the apparent metastability of the ordered strategy arrangements. This way we could reduce the transient time necessary for the slow domain growing process that forms ordered phases if the system is started from a random initial state. However, the application of the latter, slower method was unavoidable when we wished to determine the critical temperature of the first-order transition between the Potts and Ising phases, which turned out to be $K_{P-I}^{\left(\mathrm{MC}\right)}=0.970\left(5\right)$ for α=1.01.

**Figure 1:**
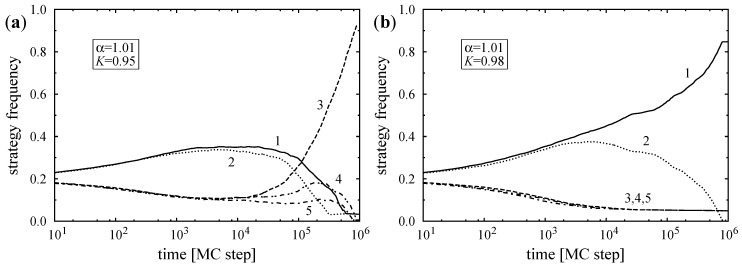
Time evolution of strategy frequencies (**a**) below and (**b**) above the Potts–Ising transition temperature $K_{P-I}^{\left(\mathrm{MC}\right)}=0.97$. Both Monte Carlo simulations were started from a random initial state for L=1,600. The labels refer to strategies.

To illustrate the domain growing process, we have performed some time-consuming simulations starting from random initial strategy distributions near the transition point. Figure 1 shows the time evolution of strategy frequencies $\rho_{i}\left(t\right)$ below and above the critical point, respectively. To suppress the effect of fluctuations in $\rho_{i}\left(t\right)$, the plotted results were averaged over a suitable period of time (typically from 0.95t to 1.05t). In the beginning, the number of players following Ising strategies rises, and the proportion of Potts strategists decreases on both sides of the transition. In this initial period, clusters of strategies nucleate, and a domain structure emerges. Afterward, the growth and shrinking of domains governs the dynamics. Below the critical temperature (see Figure 1a) the Potts domains invade Ising domains whose sizes diminish, and eventually one of the Potts clusters percolates and finally prevails in the system. Above the critical temperature (see Figure 1b), one of the Ising strategies becomes dominant following a very similar mechanism.

## 4. Results and Discussion

First, we sketch the general features that are already captured by mean-field approximation, and then move on to highlight finer details revealed by pair approximation and Monte Carlo simulations. From the Monte Carlo results, we extract the critical exponents of the system’s order-disorder phase transitions. Finally, we argue that the first-order transitions between the ordered phases are driven by entropy.

**Figure 2:**
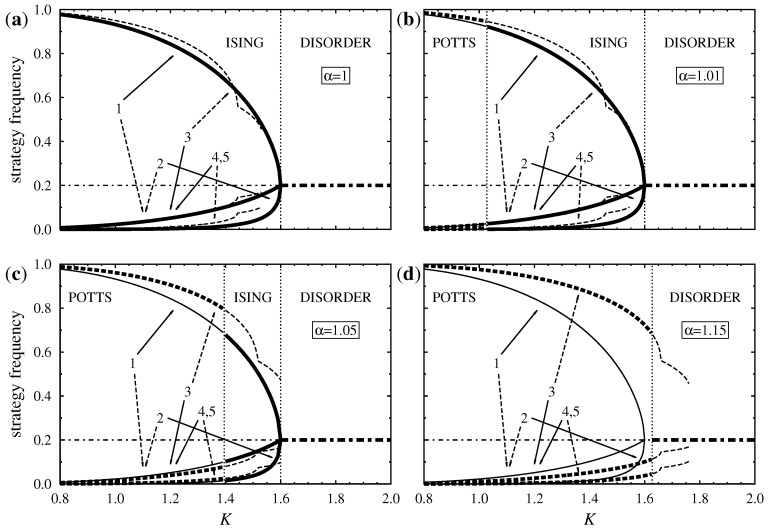
Strategy frequencies as a function of *K* as predicted by mean-field theory, when (**a**) α=1, (**b**) α=1.01, (**c**) α=1.05, and (**d**) α=1.15. Solid, dashed, and dash-dotted lines correspond to Ising, Potts, and disordered solutions, respectively. Labels and arrows help identify curves that belong to different strategies, and thick lines indicate the state with the highest free energy.

Figure 2a shows the equilibrium strategy frequencies derived within the framework of mean-field approximation when the Ising and Potts components are equally strong (α=1). Here the curves represent all possible solutions of Equation (10), stable solutions are distinguished by thick lines. This two-player game has five equal-payoff Nash-equilibria and the multi-agent system has five degenerate ground states, one for each pure strategy when the game is played on a square lattice. Henceforth, as mentioned earlier, we assume that in the two-fold degenerate Ising phase strategy 1 dominates the system at low noises ($\rho_{1}\left(K\right)\to 1$ if K→0) while strategy 3 plays the same role among the three-fold degenerate Potts phases.

Figure 2a illustrates that the thermodynamic potential Φ of the Ising phases is greater than Φ of the Potts phases at all temperatures. Raising the noise level lowers the population gap between majority and minority strategies in the ordered states, the Ising state loses its stability at a critical point $K_{I}^{\left(\mathrm{mf}\right)}=8/5=1.6$, and above this critical noise level the system evolves into a disordered phase, where all five strategies are present with the same probability, that is, $\rho_{i}=0.2$. Notice, that the latter disordered strategy distribution also exists at low noises as an unstable solution. We note, furthermore, that mean-field theory predicts the presence of a first-order discontinuity in the Potts phase which is observed neither in the dynamical pair approximation approach nor in Monte Carlo simulations.

Evidently, the increase of α stabilizes the Potts phases and modifies their noise-dependence as illustrated in Figure 2b–d. According to mean-field theory the Potts phases are stable at low noises if α=1.01 or 1.05 (see Figure 2b,c), and these ordered states transform into one of the Ising phases at $K_{P-I}^{\left(\mathrm{mf}\right)}$. The ordered Ising phase exhibits similar behavior as above and transforms continuously into the disordered phase at $K_{I}^{\left(\mathrm{mf}\right)}$ if *K* is increased. The phase transition between the Potts and the Ising phases is of the first order and it is driven by the higher entropy of the Ising phases.

In agreement with expectations, $K_{P-I}^{\left(\mathrm{mf}\right)}$ increases monotonically with α, moves toward $K_{I}^{\left(\mathrm{mf}\right)}$, and shrinks the width of the temperature region where the Ising phase is stable. Eventually, at $\alpha =\alpha_{c}^{\left(\mathrm{mf}\right)}=1.130\left(5\right)$, the predicted critical noise $K_{P-I}^{\left(\mathrm{mf}\right)}$ reaches $K_{I}^{\left(\mathrm{mf}\right)}$, and the Ising phase disappears. For $\alpha \geq \alpha_{c}^{\left(\mathrm{mf}\right)}$ the system has only one type of ordered phases and one first-order transition, as pictured in Figure 2d.

**Figure 3:**
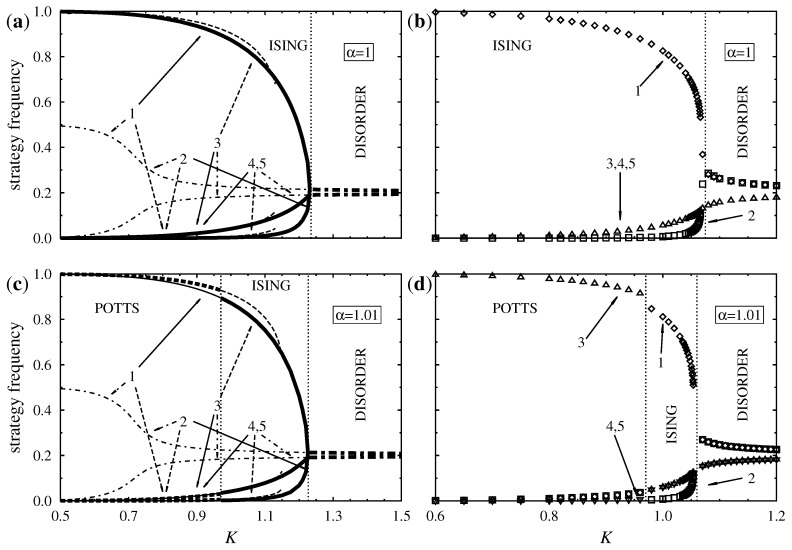
Pair approximated and Monte Carlo simulated strategy frequencies plotted against temperature: (**a**) Pair approximation for α=1, (**b**) Monte Carlo simulations for α=1, (**c**) pair approximation for α=1.01, (**d**) Monte Carlo simulations for α=1.01. In (**a**,**c**) the same notations are used as in Figure 2. In (**b**,**d**) diamonds and boxes correspond to Ising and triangles to Potts strategies.

Mean-field approaches neglect the correlations between (fixed) neighbors, but their predictions may be applied to well-mixed populations. The common feature of the present games is that the average potential is zero in the disordered phase at the level of the mean-field approximation. Moreover, in the Ising (Potts) phase the Potts (Ising) strategies do not contribute to the average potential. Pair approximation, however, takes into consideration the relevant nearest neighbor correlations and gives more adequate predictions, as shown by comparisons with Monte Carlo data in Figure 3.

The predictions of mean-field theory are justified by both pair approximation and Monte Carlo simulations at low temperatures that typically cause point defects in the ordered phases. The latter methods, however, predict a differently structured disordered phase where Ising strategies are chosen slightly more often than Potts strategies due to the correlations favoring the choice of identical neighboring strategies. It is worth mentioning that in the analytic continuation of the disordered solutions the equal frequencies of Potts strategies tend to zero as K→0.

In general, cluster variation methods overestimate the critical temperatures of continuous order-disorder transitions but give a more accurate approximation of first-order transition points between ordered phases. This expectation is confirmed by comparing the plots of Figure 3. The effects of long range correlations and fluctuations become relevant in the vicinity of the critical point and modify the power law behavior as illustrated by these results [43].

**Figure 4:**
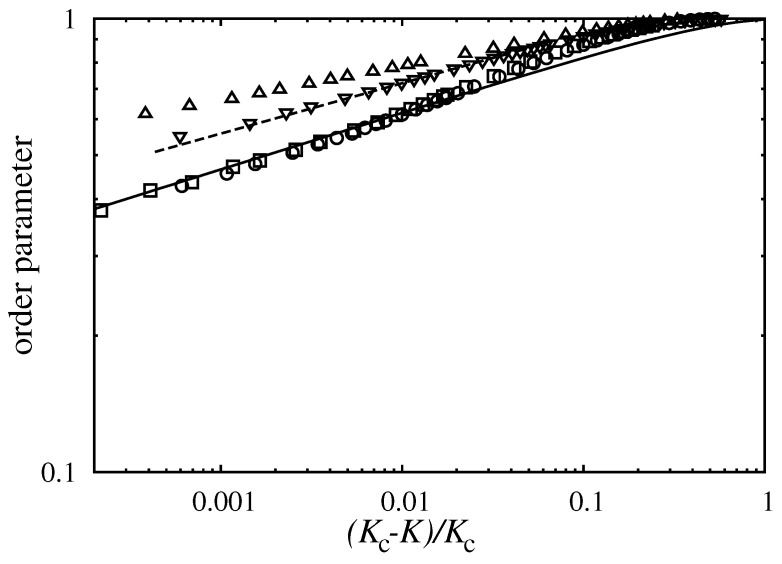
Log-log plot of order parameters versus the relative temperature. Upward and downward pointing triangles correspond to $\rho_{3}-\left(\rho_{4}+\rho_{5}\right)/2$ for α=1.02 and 1.1. Circles and boxes depict Ising magnetization data ($\rho_{1}-\rho_{2}$) for α=1 and α=1.01, respectively. For comparison, Onsager’s exact results [18] were also plotted using a solid line while the theoretical prediction for the Potts model is indicated by a dashed line.

In order to characterize the continuous transitions, we have investigated the critical algebraic behaviors hidden in the quantities $\rho_{i}\left(K\right)$. Figure 4 shows Monte Carlo simulation data for the Ising magnetization $\rho_{1}\left(K\right)-\rho_{2}\left(K\right)$ if α=1 and 1.01. For the quantitative analysis of the vanishing Potts phase we used a similar order parameter, $\rho_{3}-\left(\rho_{4}+\rho_{5}\right)/2$, which vanishes continuously at the critical point for α=1.02 and 1.1 [37,44]. All four sets of Monte Carlo data seem to follow power laws. The critical exponents were estimated by fitting algebraic functions to the relevant data points closest to their respective critical temperatures. The estimated critical exponent (β) for the Ising magnetization turned out to be $\beta_{I}=0.135\left(10\right)$ [$K_{c}=1.06665\left(10\right)$] for α=1 and $\beta_{I}=0.131\left(10\right)$ [$K_{c}=1.05373\left(10\right)$] for α=1.01 that convincingly approximates the theoretical result (β=1/8) obtained by Onsager [18] for the two-dimensional Ising model. These results support the robustness of Ising-type behavior in composite games with independent elementary coordination components (cf. Reference [15,16] for a counterexample). If α=1.1, the critical exponent for $\rho_{3}-\left(\rho_{4}+\rho_{5}\right)/2$ was found to be $\beta_{P}=0.102\left(10\right)$ [$K_{c}=1.1777\left(10\right)$] that is remarkably close to β=1/9 characteristic to the two-dimensional three-state Potts model [37]. For α=1.02, however, the Potts phase transforms into the disordered one with a decidedly different exponent, namely $\beta_{P}=0.0754\left(10\right)$ at $K_{c}=1.0432\left(10\right)$.

**Figure 5:**
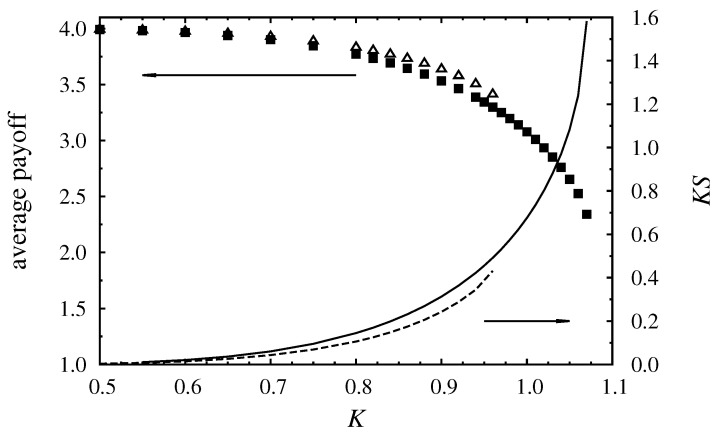
Monte Carlo results for the average payoff (symbols) and KS (lines) versus *K* if α=1. Filled boxes and the solid line correspond to the stable Ising state, empty triangles and the dashed line belong to the metastable Potts state. KS was estimated using the mean-field approximation entropy formula as $K\sum_{i}\rho_{i}\ln\rho_{i}$. Note that the average payoff is twice the average potential.

Why is the Ising state still preferred near the order-disorder transition point when $\alpha <\alpha_{c}$? Why is its free energy still higher than that of the Potts state? Figure 5 clearly shows that the contribution of entropy (KS) to the free energy has a very important role in stabilizing the Ising state. In both ordered states there are four minority strategies that are distributed differently in the two cases. The Potts phase has two higher- and two lower-frequency minority states as opposed to the three to one ratio observed in the Ising phase. Consequently, when compared at a fixed noise level, the Ising phase is less ordered, thus its entropy is higher and also grows faster as the noise level approaches $K_{I}$ from below; hence the free energy of the Ising phase grows faster and overtakes that of the Potts state. This phenomenon creates a kind of social dilemma insofar as the Potts phase with its higher average payoff is not the system’s stable equilibrium steady state. In other words, the presence of a competing higher-entropy state can prevent the community from maximizing its total payoff.

The stabilization effect of entropy can also be observed in elementary coordination games [15,45] that form a basis in the subspace of coordination-type games [12]. An *n*-strategy elementary coordination game is an extension of the original coordination game [3,9,46,47,48] in which beside two coordinated strategies n−2 additional neutral strategies are also available, which provide zero payoff regardless of the opponent’s strategy. Notice that this game is exactly the Ising component of the game defined by the payoff matrix in Equation (2). If this game is played on a square lattice, then the system undergoes an order-disorder phase transition as the noise level is increased across a critical value. Below the transition point the frequency of one of the coordinated strategies starts to grow, while the number of players following other strategies is reduced. The proportion of players following the other coordinated strategy drops faster than the proportion of those who play one of the neutral strategies. In the disordered phase all strategies are similarly frequent, both coordinated strategies are played with equal and slightly higher probabilities than their neutral counterparts. The order and the critical noise level of this phase transition is determined by the number of neutral strategies. If *n* is greater than the threshold $n_{\mathrm{th}}=27$ [15], then the system exhibits a first-order phase transition, whereas it becomes continuous if fewer strategies are available. The more neutral strategies there are, the smaller the critical temperature turns out to be. To explain these results one should observe how increasing the number of neutral strategies affects the entropy content in the two phases. Deep within the ordered phase, it is easy to see that the competing high-entropy state’s thermodynamical potential can grow beyond limit at a fixed noise level as more and more additional strategies are introduced. The ordered state may maintain its stability by either increasing its average payoff via further homogenization, which decreases entropy and is capped by the limiting case of uniform cooperation, or by raising its entropy through dismantling order that in turn diminishes average payoff and drives the system toward the disordered phase. As a result, the stability region of the high-entropy disordered phase becomes larger when additional neutral strategies are introduced into an elementary coordination game. A similar entropy-based stabilization is characteristic of high-entropy alloys [49,50,51], a promising family of materials for several technical purposes.

If decoupling of independent game components holds at the mean-field level, then the above-described results should give us an idea about what happens in games made up of an Ising and an (n−2)-strategy Potts subgame, just like the one in Equation (2). We should expect to see competing Ising and Potts phases for low enough temperatures and the entropic stabilization of the Ising state near the transition temperature when α is not too high. As *n* is increased, critical temperatures should become lower because of the faster increase in the entropy content of the disordered phase.

## 5. Conclusions

In this paper, we have studied a five-strategy evolutionary potential game that pits an Ising-type and a three-state Potts-type coordination game against each other. This interaction combines the two games as independent subgames of the first two and last three strategies, respectively. The application of the logit strategy update rule has allowed us to utilize various well-known concepts and methods of statistical physics. The systematic investigation of the spatial system is restricted to a model where the players are located at the sites of a square lattice and repeatedly play the five-strategy game against their nearest neighbors. Due to this choice, the observed properties of the stationary states can be compared with universal features characteristic of the original Ising and Potts models.

The game is defined by a single parameter α quantifying the ratio of payoffs in the ordered Ising and Potts phases. It is found that both of the two degenerate Ising phases are stable at low noises if α≤1. Their higher entropy favors the Ising phases over the Potts phases if α=1. The contribution of entropy, however, vanishes at low noises, therefore the advantage of the Ising phases can be compensated if the Potts phases provide slightly higher payoffs (α>1). In the latter cases one can observe a first-order phase transition from the Potts phase to the Ising phase if the noise level (temperature) is increased. The critical noise level of this first-order transition increases with α−1 until eventually the Ising phase (along with the first-order Potts–Ising transition) disappears because the Ising phase loses its metastability to the disordered state before the Potts phase transforms into the same disordered phase.

Independently of the preferred ordered phases at low noises, the system undergoes a continuous (critical) order-disorder phase transition. The quantitative analysis of the order parameters indicates that in most of the cases these continuous transitions preserve the universal behavior of the corresponding (Ising or Potts) model. Additionally, we have observed non-universal behavior in a narrow range of parameters where the interplay between these two different transitions seems to be relevant. It is worth mentioning that similar non-universal critical phase transitions were also reported in several versions of the Ashkin-Teller model [32,52,53,54] which are also combinations of elementary coordination games [16]. The systematic analysis of the non-universal phase transitions goes beyond the scope of the present work. At the same time, we think that the concept of matrix decomposition could prove to be a general frame for the identification of multi-dimensional order parameters as well as for the parametrization of interactions when the tensor renormalization group approach [55,56] is used for the investigation of critical phase transitions.

We have found that the first-order transition from the Potts state to the Ising state is accompanied by a loss of average income for α>1. The stability of the Ising phase resembles the tragedy of the commons, where the community as a whole does not maximize its total payoff. In this situation the social dilemma is caused by the higher entropy content of the Ising state. In other words, the number of microscopic states in the Ising phase significantly exceeds those which belong to the Potts phase. It is expected that a similar phenomenon can occur in many other social systems where the increasing number of noise-dependent mistakes in the competing ordered phases can compensate for the advantage of higher average payoffs.
